# Heterogeneous immune landscapes and macrophage dynamics in primary and lung metastatic adenoid cystic carcinoma of the head and neck

**DOI:** 10.3389/fimmu.2024.1483887

**Published:** 2024-12-04

**Authors:** Xuelian Wang, Tingyao Ma, Hongfei Liu, Shujing Zhang, Guoliang Yang, Yue Zhao, Lu Kong, Ran Gao, Xiaohong Chen

**Affiliations:** ^1^ Department of Otolaryngology, Head and Neck Surgery, Beijing Tongren Hospital, Capital Medical University, Beijing, China; ^2^ National Human Diseases Animal Model Resource Center; State Key Laboratory of Respiratory Health and Multimorbidity, National Health Commission (NHC) Key Laboratory of Comparative Medicine, Institute of Laboratory Animal Science, Chinese Academy of Medical Sciences (CAMS) and Peking Union Medical College (PUMC), Beijing, China; ^3^ Department of Biochemistry and Molecular Biology, Capital Medical University, Beijing, China

**Keywords:** adenoid cystic carcinoma, lung metastasis, tumor immune microenvironment, tumor-associated macrophage, immune evasion

## Abstract

**Introduction:**

Recurrent or metastatic adenoid cystic carcinoma (ACC) of the head and neck is rare and highly aggressive. Due to the ineffectiveness of immune checkpoint therapies, this study aims to investigate the tumor immune microenvironment of primary tumor tissues and lung metastatic tissues and to comprehend the challenges of immunotherapy.

**Methods:**

We analyzed RNA sequencing data and constructed immune landscapes from 25 primary tumors and 34 lung metastases. The data were then validated by immunohistochemistry and single-cell sequencing analysis.

**Results:**

Compared to adjacent normal tissues, both primary and lung metastatic ACC showed low immune infiltration. Lung metastases had higher immune infiltration levels and antigen presentation scores but also higher T cell exclusion and dysfunction scores. Single-cell sequencing data and immunohistochemistry revealed abundant immunosuppressive tumor-associated macrophages in lung metastases. Patients with high M2 macrophage infiltration had shorter lung metastasis-free survival.

**Discussion:**

Primary and lung metastatic ACC exhibit heterogeneous tumor immune microenvironments. Higher immune cell infiltration in lung metastases is countered by the presence of suppressive tumor-associated macrophages, which may limit effective anti-tumor responses.

## 1 Introduction

Adenoid cystic carcinoma (ACC) originates from glands and is uniquely characterized by perineural invasion and a high 5-year distant metastasis rate of up to 52% (1, 2), with the lungs being the most common site of distant metastasis (3). Aggressive surgery and postoperative radiotherapy provide only short-term local relief but fail to suppress distant metastases, resulting in a poor long-term prognosis for patients. Therefore, breakthroughs in the therapy of ACC lie in the containment of lung metastases.

Although numerous molecular targeted drugs have been preliminarily explored, overall response rates and survival benefits remain unsatisfactory (4, 5). For example, MYB fusion mutation and MYB overexpression are hallmark driving molecular events in ACC development (6), making targeting inhibitors against MYB and its downstream genes a promising approach. Mandelbaum et al. (7) found that all-trans retinoic acid (ATRA) significantly inhibits MYB expression in ACC in zebrafish models. However, a Phase II clinical trial of ATRA in advanced ACC patients showed an objective response rate (ORR) of 0% (8), and a trial of ATRA in combination with apatinib reported an ORR of only 18% (9). Aberrant activation of the Notch pathway has been demonstrated to correlate with poor prognosis in solid-type ACC and distant metastasis in patients (10, 11). Yet, γ-secretase inhibitors targeting the Notch pathway have shown limited efficacy and notable gastrointestinal side effects (12). Tyrosine kinase inhibitors (TKIs) such as sunitinib and lenvatinib can only control disease stabilization (13). Cytotoxic drugs have also shown disappointing results in recurrent and metastatic ACC (4).

The advent of immune checkpoint inhibitors (ICIs) and cell therapy has revolutionized cancer treatment paradigms (14). Antibody-mediated blockade of the PD-1/PD-L1 pathway has successfully treated a subset of patients with advanced cancers, such as melanoma, non-small cell lung cancer, renal cell carcinoma, classical Hodgkin lymphoma, and head and neck squamous cell carcinoma, and has been approved by the U.S. Food and Drug Administration (FDA) for the treatment of various cancers (15, 16). Compared to targeted therapies, ICIs can induce durable responses in patients with metastatic cancers (17). Unfortunately, the response rate of ICIs in ACC patients remains low, posing a significant challenge in identifying biomarkers for ICI response and resistance. It is reported that the ORR of PD-1 inhibitors as monotherapy for ACC ranges from 0-9% (18). In a Phase II clinical trial of nivolumab combined with ipilimumab in 32 patients with advanced ACC, only 2 patients showed confirmed partial responses (2/32, 6%) (19). Another Phase II clinical trial evaluating the efficacy of a VEGFR inhibitor and a PD-L1 inhibitor in recurrent/metastatic ACC patients reported a confirmed ORR of 18% (20). Due to the low tumor mutation burden (TMB) and typically low or absent PD-L1 expression in ACC, patients with advanced ACC are unlikely to benefit from ICIs alone (21). Previous studies indicated that primary ACC tumors are predominantly characterized by immune exclusion and immune desert phenotypes (22, 23), with over 60% classified as “cold” tumors (24). However, comprehensive analyses of the immune microenvironment in metastatic ACC have been largely overlooked. It is increasingly recognized that the metastatic tumor microenvironment exhibits heterogeneity compared to the primary site, indicating that studies focused solely on primary tumors may introduce biases in developing therapies for metastatic tumors (25). Therefore, the immune microenvironment heterogeneity between primary and metastatic ACC deserves an in-depth and comprehensive investigation.

Accordingly, our research focuses on investigating differences in the TIME between ACC lung metastases and primary tumors. We discovered that adjacent normal pulmonary tissue demonstrated more adequate immune condition due to enrichment of total innate and adaptive cells. However, both ACC lung metastases and primary tumors displayed the immunosuppressive environments. Notably, compared to primary tumors, lung metastases exhibited higher immune cell infiltration but also had a unique immunosuppressive environment which provide a niche for tumor cell colonization and growth. Subsequently, using immunohistochemistry and single-cell sequencing, we confirmed the presence of numerous immunosuppressive tumor-associated macrophages (TAMs) in the lung metastases. M2 macrophages were associated with early lung metastasis in patients and could potentially serve as biomarkers for predicting immune response. These findings offer new insights into the TIME of lung metastatic ACC and may guide future research in precision immunotherapy for metastatic ACC.

## 2 Materials and methods

### 2.1 Clinical sample collection

The experimental design and informed consent procedures were approved by the Ethics Committee of Beijing Tongren Hospital, affiliated with Capital Medical University (Ethics Approval Number: TREKY2020-021). Written informed consent was obtained from all patients participating in this study, and all procedures were conducted in accordance with the Declaration of Helsinki. Surgical procedures were performed on ACC patients who met the surgical indications, and corresponding regions of tumor center and adjacent non-tumorous tissues were collected. In this study, we collected 32 primary tumor samples and 47 lung metastasis samples, including three matched pairs from the same patients. The diagnosis of ACC was histopathologically confirmed by at least two pathologists. Histological grading was conducted based on the pathological subtypes proposed by Szanto et al. in 1984 (26): grade I for predominantly cribriform or tubular patterns, grade II for mixed patterns with cribriform and tubular components with less than 30% solid areas, and grade III for predominantly solid components exceeding 30%. The diagnostic criteria for high-grade transformation were based on the characteristic histological features proposed by Seethala et al. (27).

### 2.2 Transcriptome sequencing

Appropriate tissue samples were collected into corresponding numbered grinding tubes, and 1.5 mL of TRIzol lysis reagent was added. RNA sequencing (RNA-seq) was performed at the Beijing Genomics Institute. Total RNA was extracted using an RNA extraction kit and reverse-transcribed into cDNA using a reverse transcription kit. The concentration, RNA integrity number, 28S/18S ratio, and fragment size of total RNA were determined using the Agilent 2100 Bioanalyzer. Samples meeting quality standards were used for library construction and sequencing on an MGISEQ2000RS platform. Subsequent data analysis, visualization, and mining were performed using the Dr. Tom multi-omics data mining system (https://biosys.bgi.com). Differential gene expression analysis between groups was conducted using DESeq2 (28) with criteria of Fold Change ≥ 1 and Adjusted P value ≤ 0.05. Differentially expressed genes were functionally classified based on Gene Ontology (GO) and Kyoto Encyclopedia of Genes and Genomes (KEGG) annotations, as well as official classifications. KEGG enrichment analysis was performed using the phyper function in R software, and GO enrichment analysis was carried out using the TermFinder package. Genes with Qvalue ≤ 0.05 were considered significantly enriched in the candidate gene set.

### 2.3 Immune infiltration and functional analysis

Single-sample gene set enrichment analysis (ssGSEA) was performed using the GSVA package in R software (version 4.3.1) (29). Marker genes characterizing immune cell types were obtained from Bindea et al. (30) and Charoentong et al. (31), while angiogenesis marker genes were sourced from Masiero et al. (32). The marker gene set for MHC class I antigen presenting machinery (APM) included HLA-A, HLA-B, HLA-C, B2M, TAP1, TAP2, and TAPBP (33). The overall immune infiltration score (IIS) was defined as the average of the standardized values of innate and adaptive immune scores (33). The T-cell infiltration score (TIS) was defined as the average of the standardized values of nine T-cell subtypes (33). Cytolytic activity (CYT), an indicator of immune cell cytotoxic activity, was defined as the geometric mean of the transcriptional expression levels of the effector genes GZMA and PRF1 (34). Tumor Immune Dysfunction and Exclusion (TIDE) is a computational method based on transcriptomics that simulates two major mechanisms of tumor immune evasion: T-cell dysfunction and T-cell exclusion. It can predict cancer patient response to immune checkpoint inhibitors (35). Batch-corrected normalized data were input into the TIDE website (http://tide.dfci.harvard.edu) for calculation. Additionally, the CIBERSORT algorithm, based on deconvolution, was used for relative quantification analysis of immune cell proportions (36).

### 2.4 Single-cell sequencing data analysis

The previously generated single-cell sequencing dataset used in this study has been uploaded to the public repository Gene Expression Omnibus (GEO) under the accession number GSE216852. This dataset includes single-cell 3’-RNA sequencing data from one primary ACC patient and one lung metastasis ACC patient. The Seurat R package was utilized to analyze the ScRNA-seq data according to standard analysis procedures. After log normalization and dimensionality reduction of the differential gene expression data of macrophage populations, the cells were divided into six clusters. Uniform Manifold Approximation and Projection (UMAP) plots, violin plots, and bubble plot analysis results were generated using the Monocle 2.0 package.

### 2.5 Immunohistochemistry

Paraffin-embedded tissue sections (4 μm thick) were deparaffinized in fresh xylene and subjected to antigen retrieval. Endogenous peroxidase activity was quenched by covering the sections with 3% hydrogen peroxide for 15 minutes. Tissue sections were blocked with 10% goat serum at 37°C for 1 hour, then incubated overnight at 4°C with primary antibodies against CD8 (ZSGB-Bio, ZA-0508), CD4 (ZSGB-Bio, ZA-0519), and CD68 (PTM-BIO, PTM-5130). Detection of horseradish peroxidase (HRP) activity was performed using the PV-6000D immunohistochemistry kit (ZSGB-Bio). Sections were counterstained with hematoxylin, differentiated with hydrochloric acid, dehydrated, and mounted. Rabbit or mouse monoclonal IgG was used as a negative control. Images were captured using the Pannoramic^®^ 250 FLASH scanner. The number of positive cells per unit area was quantified using Image J software (version 13.0.6, National Institutes of Health, USA).

### 2.6 Statistical analysis

Data were analyzed using GraphPad Prism software (version 9.5.1, GraphPad Software, CA, USA) and R software (version 4.3.1). The Mann-Whitney U test was used to compare the median distributions between two sample groups. Spearman correlation analysis was employed to assess the correlations between different indices. Lung metastasis-free survival was estimated using the Kaplan-Meier method, and statistical differences were determined using the Log-rank test. Statistical significance was indicated as follows: P<0.05 (*), P<0.01 (**), P<0.001 (***), P<0.0001 (****).

## 3 Result

### 3.1 Overall immune infiltration analysis

**Figure 1:**
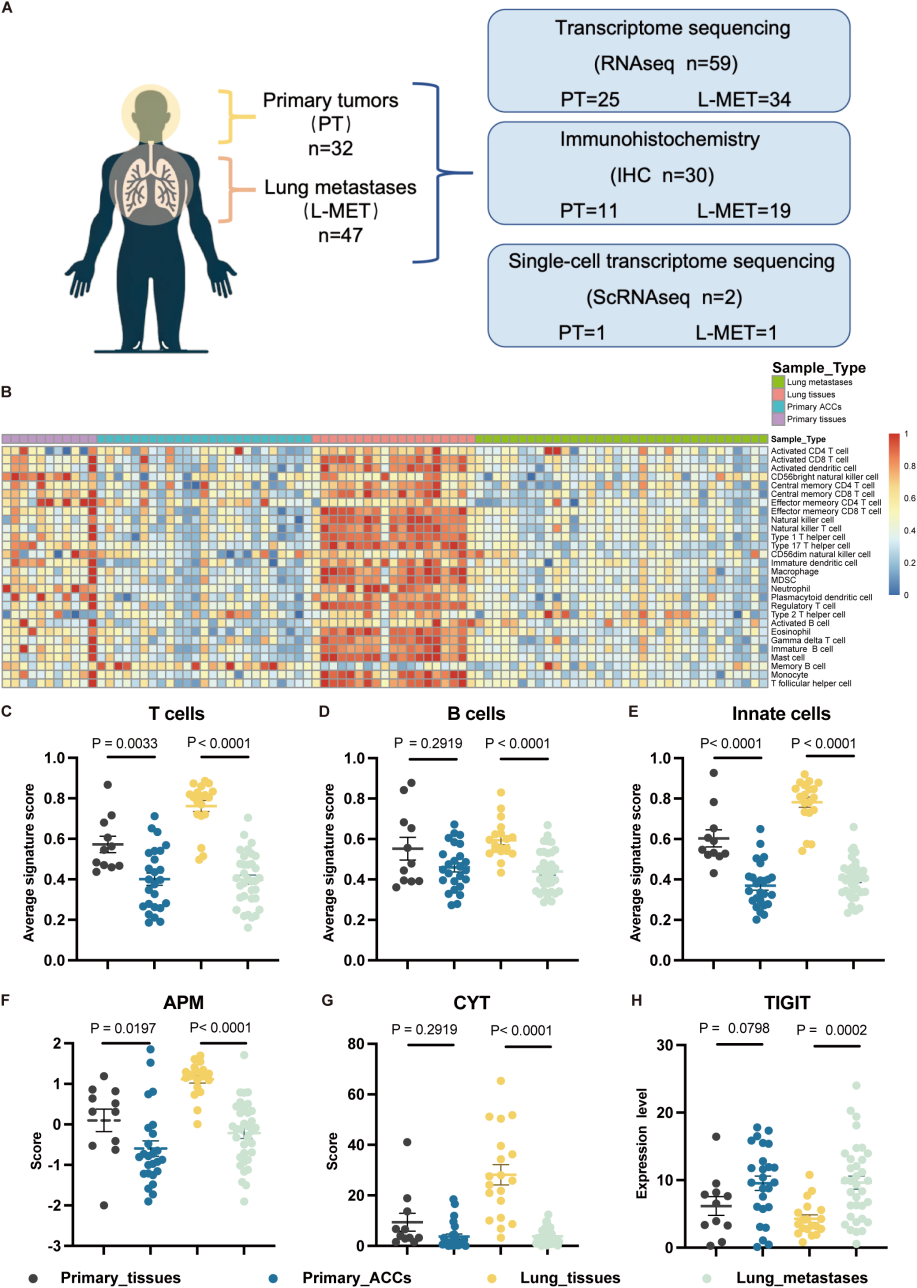
Study overview and overall immune infiltration analysis based on RNA-seq data. **(A)** A cohort of 79 patients with adenoid cystic carcinomas and available tumor was interrogated for immune landscapes characterization. **(B)** Heatmap of 28 types immune cell scores based on ssGSEA in different samples, and all cell types are defined by known marker genes (see **Supplementary Table S2**). **(C, D)** The statistical plots showing the average T cells **(C)**, B cells **(D)** and innate cells **(E)** signature score in the adjacent non-tumor primary tissues (black), primary ACCs (blue), lung tissues (yellow) and lung metastases (green). **(F, G)** The statistical plots showing the antigen presenting machinery (APM) score **(F)**, cytolytic activity (CYT) score **(G)**. **(H)** The statistical plot showing the mRNA expression level of TIGIT. The p values in **(C-H)** were calculated using the two-tailed Mann–Whitney U test. Data are presented as the mean ± SEM.

To elucidate the heterogeneity in the tumor immune microenvironment (TIME) between primary tumors (PT) and lung metastases (L-MET) of ACC, we conducted a comprehensive analysis of both by transcriptome sequencing, single-cell sequencing, and immunohistochemistry (**Figure 1A**). The basic clinical characteristics of the cohort are summarized in **Supplementary Table S1**. Transcriptome sequencing was performed on 25 primary tumor samples and 11 matched adjacent non-tumor primary tissues, as well as 34 lung metastasis samples and 19 matched normal lung tissues. Single-sample gene set enrichment analysis was conducted based on the characteristic gene sets of 28 immune cell types. The heatmap displayed the abundance of various immune cells across all samples, revealing distinct differences between different sample types (**Figure 1B**). Adjacent normal lung tissues exhibited a richer immune contexture due to the enrichment of T cells, B cells, and innate cells (**Figures 1C–E**). Both lung metastases and primary tumors displayed relatively low immune infiltration environments. Additionally, the APM and CYT score of tumor samples were lower than those of adjacent normal tissues, accompanied by elevated expression of the immune checkpoint TIGIT (**Figures 1F–H**). Overall, the abundance and functions of immune cells in the TIME of primary and metastatic lesions were significantly lower than those in adjacent normal tissues.

### 3.2 Heterogeneity immune landscapes between primary ACCs and lung metastases

**Figure 2:**
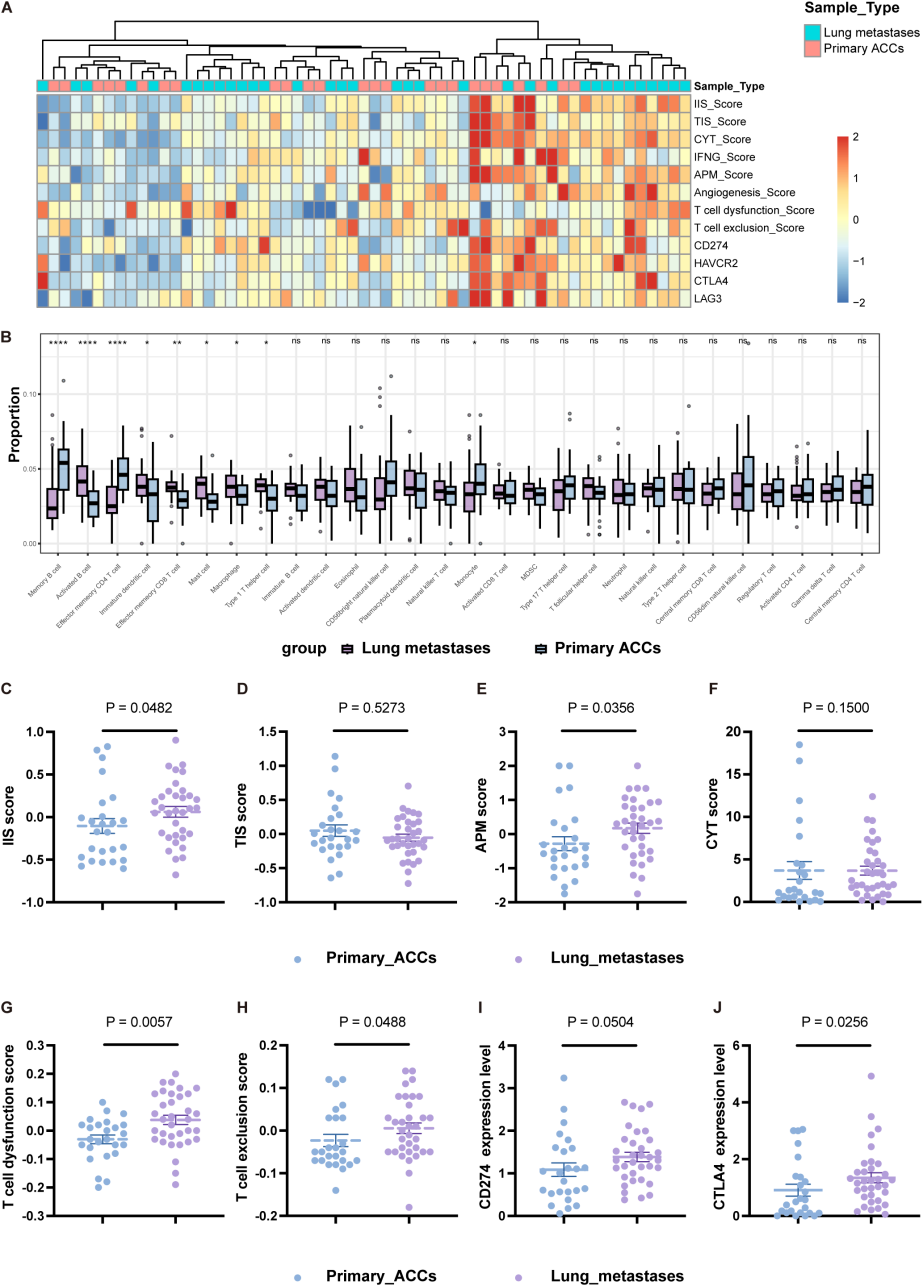
Heterogeneity immune landscapes between primary ACCs and lung metastases. **(A)** Heatmap showing indicators that characterize the activation and suppression of immune function in primary ACCs and lung metastases. **(B)** Box-plot of the relative proportion of 28 types of infiltrating immune cells. **(C-F)** The statistical plots showing the IIS **(C)**, TIS **(D)**, APM **(E)**, CYT **(F)** score between primary ACCs and lung metastases. **(G, H)** The statistical plots show the T cell dysfunction **(G)** and exclusion **(H)** score. **(I, J)** The mRNA expression level of CD274 **(I)** and CTLA4 **(J)** between primary ACCs and lung metastases. The p values in **(B-J)** were calculated using the two-tailed Mann–Whitney U test. Data are presented as the mean ± SEM. P<0.05 (*), P<0.01 (**), P<0.001 (***), P<0.0001 (****), and P>0.05 (ns).

Next, we compared the differences in immune cell types, functional activation, and inhibition levels between primary tumors and lung metastases, revealing a highly distinct immune landscape (**Figures 2A, B**; **Supplementary Figure S1A**). The IIS was higher in L-MET than in PT (**Figure 2C**). Although the TIS showed no significant difference (**Figure 2D**), the composition of specific immune lineages varied (**Figure 2B**). Effector memory CD4 T cells were significantly more abundant in PT (P<0.0001), while effector memory CD8 T cells were significantly less abundant in PT compared to L-MET (P<0.01). Certain immune cell infiltrations, such as activated B cells, effector memory CD8 T cells, and Th1 cells, were markedly higher in lung metastases, suggesting a stronger adaptive immune response. Conversely, immunosuppressive immature dendritic cells and macrophages were also more prevalent in L-MET and positively correlated with activated CD8 T cells, natural killer (NK) cells, and natural killer T (NKT) cells (**Figure 2B**; **Supplementary Figure S1B**). The anti-tumor immune response partially depends on the antigen-presenting capacity. Our results showed that the APM score was significantly higher in L-MET compared to PT (P<0.05) (**Figure 2E**). The expression of HLA class I molecules and HLA class II molecules was significantly higher in L-MET (**Supplementary Figures S2A–G**). However, there was no substantial difference in CYT score between PT and L-MET (P=0.15) (**Figure 2F**), suggesting additional immunosuppressive mechanisms may exist in lung metastases. We further analyzed T-cell dysfunction and T-cell exclusion scores, finding that both scores were significantly higher in L-MET (P<0.05) (**Figures 2G, H**). The expression of the immune checkpoint molecule CTLA4 was also higher (P<0.05). CD274 (encoding PD-L1) expression was slightly higher in lung metastases, though not statistically significant (P=0.0504).

In summary, our study reveals heterogeneity in the tumor immune microenvironment between primary head and neck ACC and its lung metastases. The differences in immune cell composition, immune responses function, and inhibitory mechanisms between lung metastases and primary tumors are substantial. These differences should be carefully considered when designing preclinical drug development studies targeting lung metastases.

### 3.3 Macrophages infiltration as a core feature of lung metastasis

**Figure 3:**
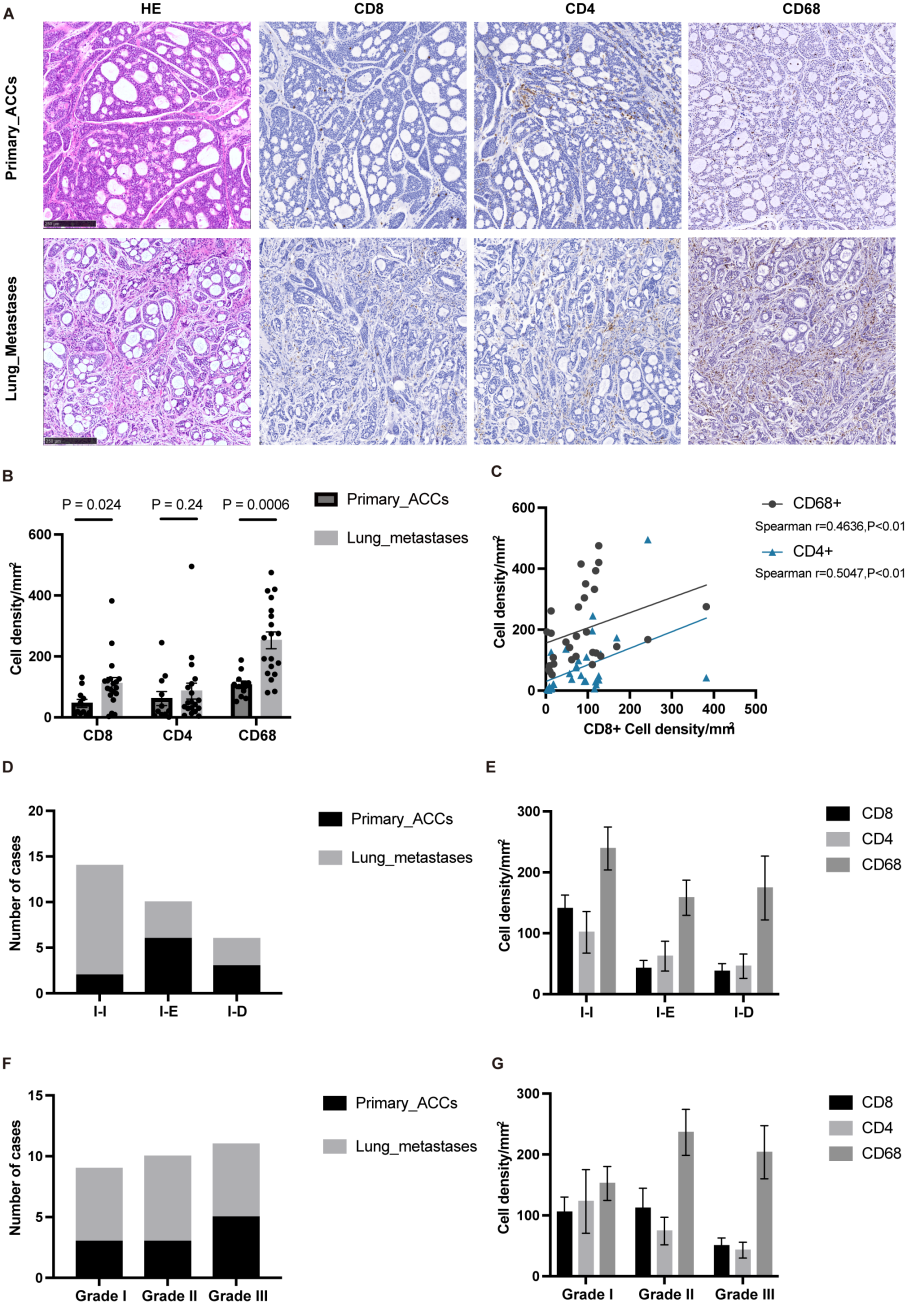
IHC confirmed that macrophages infiltration was dominant in lung metastases. **(A)** The typical histopathological features of the primary ACCs and lung metastases. **(B)** The statistical plot showing the CD8+, CD4+, CD68+ cell density per mm2. **(C)** Correlation between the cell density of CD8 positive cells and the cell density of CD4 and CD68 positive cells. **(D)** TIME classification of primary ACCs and lung metastases. **(E)** The cell density of CD8, CD4 and CD68 positive cells in different TIME type tumor tissues. **(F)** Histopathologic classification of primary ACCs and lung metastases. **(G)** The cell density of CD8, CD4 and CD68 positive cells in different histopathologic type tumor tissues. The p values were calculated using the two-tailed Mann–Whitney U test and Spearman correlation analysis. Data are presented as the mean ± SEM.

Our transcriptome sequencing analysis revealed that the overall immune cell infiltration is higher in lung metastases compared to primary tumors. However, the TIME also exhibits elevated immune suppression mechanisms in L-MET. To further validate the RNA-seq results and explore the specific cell types exerting immunosuppressive effects, we performed immunohistochemical validation on 30 tumor samples. Consistent with the sequencing results, the number of CD8+ and CD68+ positive immune cells infiltrating lung metastases was significantly higher than in primary tumors (P<0.05 and P<0.01, respectively) (**Figures 3A, B**). We found a remarkable enrichment of macrophages in the stroma of lung metastases (**Figure 3A**), which were positively correlated with CD8+ T cells (Spearman r=0.4588, P<0.01) (**Figure 3C**). Tumor-associated macrophages typically have immunosuppressive and pro-tumorigenic roles in many cancers (37). These findings suggest that macrophages may be a critical component of the immunosuppressive microenvironment in lung metastases of ACC.

Tumor immune phenotypes are often correlated with malignant progression and response to immunotherapy (38, 39). Based on the spatial distribution of T-cell infiltration, TIME can be classified into three types: immune-inflamed (I-I), immune-excluded (I-E), and immune-desert (I-D) (38). We examined the TIME classification in PT and L-MET, finding that lung metastases predominantly exhibited the I-I phenotype (**Figure 3D**), which is consistent with our previous findings (40). CD68+ immune cells were predominant across different TIME classifications (**Figure 3E**). The distribution of different histological grades in PT and L-MET samples was relatively uniform (**Figure 3F**), with higher densities of CD68+ immune cells in Grade II and Grade III tissues containing solid components (**Figure 3G**). Solid components are a key indicator of poor prognosis in ACC patients and are closely associated with distant metastasis (10, 41). Further investigation is needed to determine whether the malignant progression of solid-type tumors is related to tumor-infiltrating macrophages.

### 3.4 Tumor-associated macrophages are predominantly immunosuppressive

**Figure 4:**
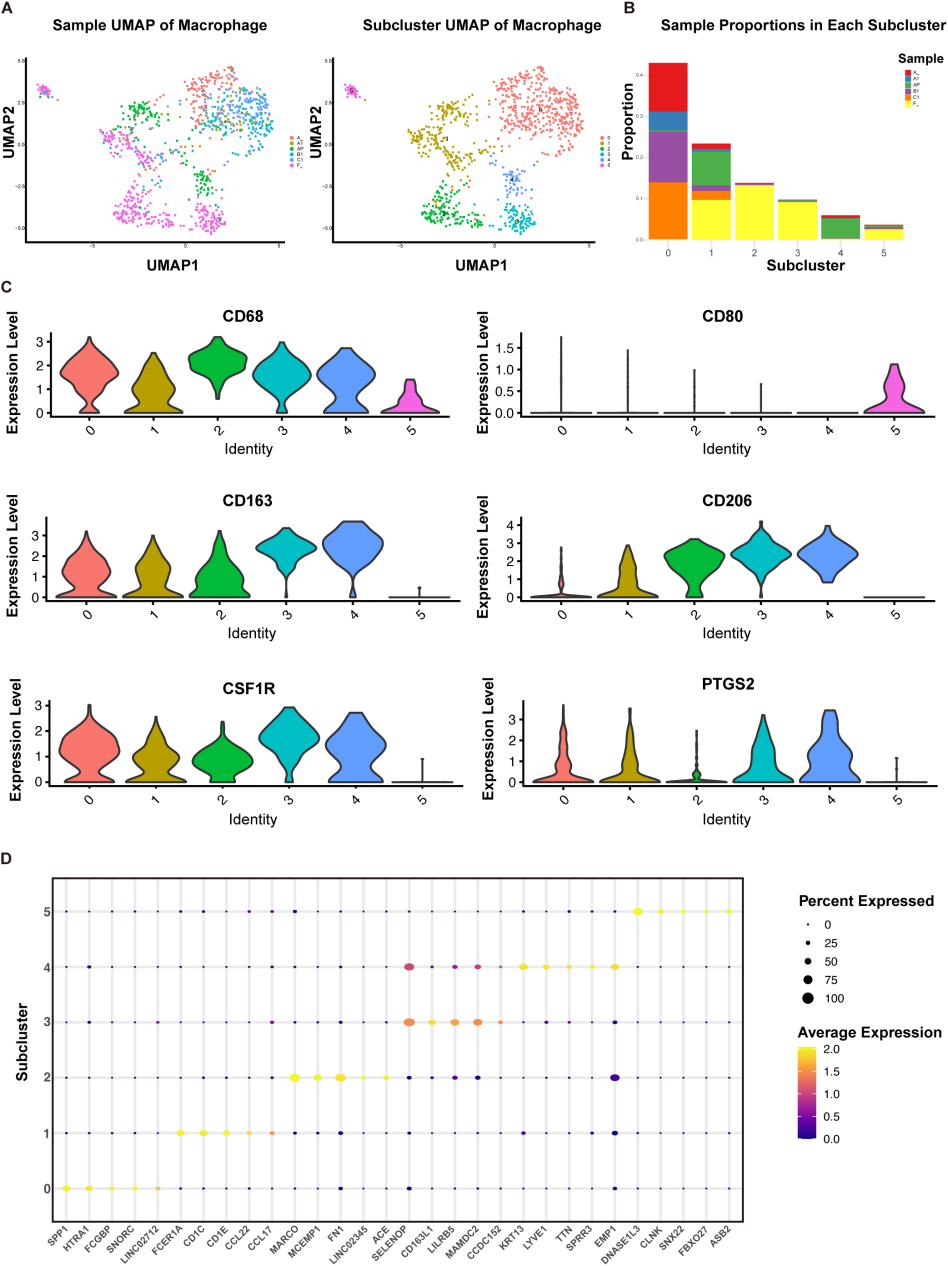
Subpopulation analysis of macrophages based on single-sell sequencing. **(A)** UMAP plot of 964 macrophages from primary ACC (A), adjacent primary tissue (AP), lung metastases (A1, B1, C1), and normal lung tissues (F). **(B)** Proportions of each sample type within each cell cluster. **(C)** Violin plots displaying the expression of specific genes in each cell type cluster. **(D)** Bubble plot displaying the top 5 highly expressed genes in each cell type cluster.

Tumor-associated macrophages exist in various subtypes, some of which have been shown to correlate with cancer prognosis and resistance to immunotherapy (42). To further investigate the subtypes and functions of macrophages in ACC, we utilized previously published single-cell sequencing data from our team (43). We performed single-cell sequencing on a primary tumor (A) and matched adjacent non-tumor tissue (AP), as well as on a set of lung metastases of different sizes (A1, B1, C1) and matched adjacent lung tissue (F), identifying macrophage populations (**Figure 4A**). Further sub-clustering revealed six macrophage types, with cluster 0 identified as tumor-infiltrating macrophages, and clusters 1 to 4 primarily representing macrophages from adjacent tissues (**Figure 4B**). Analysis of specific macrophage markers showed that cluster 5 highly expressed the M1 marker CD80, while clusters 0 to 4 predominantly expressed M2 markers CD163, CD206, CSF1R, PTGS2 (**Figure 4C**). We further analyzed the top five highly expressed genes in each macrophage cluster (**Figure 4D**), most of which were related to immunoregulatory functions and tissue repair of macrophages. Genes such as MARCO, FN1, ACE, and CD163L1 are likely associated with the immunoregulatory functions of M2 macrophages, which typically promote tissue repair, anti-inflammatory responses, and support tumor growth (44, 45). Cluster 5 highly expressed DNASE1L3, a gene involved in DNA degradation and potentially in the clearance of self-antigens during cell death processes (46). While cluster 5 may act as anti-tumor macrophages, they represent only a small fraction.

Overall, our IHC and ScRNA-seq analyses confirmed that the predominant cell population in the TIME of ACC consists of M2 macrophages, especially in lung metastases, where they exhibit significant immunosuppressive functions.

### 3.5 Immune landscape differences among subgroup populations grouped by TAMs

**Figure 5:**
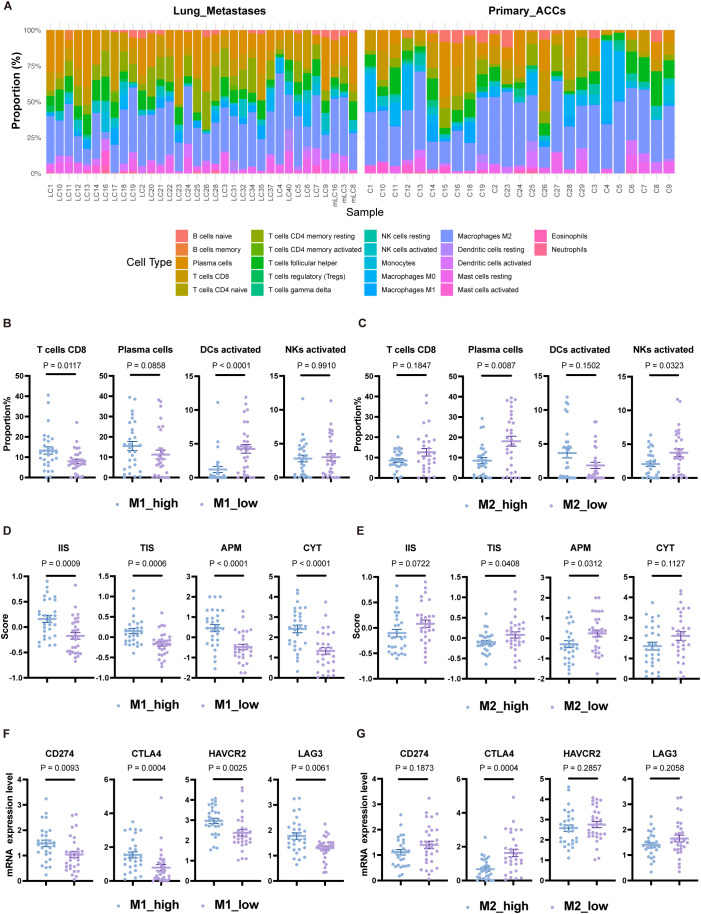
Immune landscape differences among subgroup populations grouped by TAMs. **(A)** Relative proportions of 22 immune cell types in each sample based on the CIBERSORT algorithm. **(B)** Proportions of CD8 T cells, plasma cells, activated DCs, and activated NK cells grouped by the median proportion of M1 macrophages. **(C)** Proportions of CD8 T cells, plasma cells, activated DCs, and activated NK cells grouped by the median proportion of M2 macrophages. **(D)** Scores for IIS, TIS, APM, and CYT grouped by the median proportion of M1 macrophages. **(E)** Scores for IIS, TIS, APM, and CYT grouped by the median proportion of M2 macrophages. **(F)** Expression levels of CD274, CTLA4, HAVCR2, and LAG3 grouped by the median proportion of M1 macrophages. **(G)** Expression levels of CD274, CTLA4, HAVCR2, and LAG3 grouped by the median proportion of M2 macrophages. The p values were calculated using the two-tailed Mann–Whitney U test. Data are presented as the mean ± SEM.

We performed a relative quantification analysis of immune cells using the CIBERSORT deconvolution algorithm (36). Consistent with previous results, macrophages were the major component of the myeloid immune cell subpopulation, with M1 macrophages representing a small fraction and M2 macrophages predominating in both primary and lung metastatic lesions (**Figure 5A**). Among the three pairs of matched primary and lung metastatic tumors, two cases showed significant amplification of M2 macrophages in lung metastases, while one case showed a significant increase in plasma cells (**Supplementary Figure S3A**).

Based on the relative proportions of M1 and M2 macrophages, we categorized the population into M1_high, M1_low, M2_high, and M2_low groups according to their respective medians. The M1_high group had higher CD8 T-cell levels compared to the M1_low group (P<0.05), but lower levels of activated dendritic cells (DCs) (P<0.0001) (**Figure 5B**). The proportion of M1 macrophages showed a positive correlation with CD8 T-cell proportion (Spearman r=0.4358, P<0.001) and a negative correlation with activated DCs (Spearman r=-0.6436, P<0.0001) (**Supplementary Figure S3B**). The M2_low group exhibited significantly higher levels of plasma cells and activated NK cells compared to the M2_high group (P<0.05), though activated DCs were lower in the M2_low group, albeit not significantly (P=0.1502) (**Figure 5C**). These findings suggest that the reduction of activated DCs may represent another immune escape mechanism in patients with an anti-tumor TAM phenotype. In line with the anti-tumor function of M1 macrophages, the M1_high group had elevated IIS, TIS, APM scores, and CYT scores compared to the M1_low group (all P<0.001) (**Figure 5D**), indicating a stronger immune response. However, the expression levels of immune checkpoint molecules CD274, HAVCR2, CTLA4, and LAG3 were also higher in the M1_high group (P<0.01) (**Figure 5F**). The M2_low group showed higher TIS and APM scores compared to the M2_high group (all P<0.05), but there was no significant difference in CYT scores (P=0.1127) (**Figure 5E**). CTLA4 expression was higher in the M2_low group (P<0.001) (**Figure 5G**).

In conclusion, categorizing ACC patients based on macrophage transcript levels revealed differences in their immune landscape, including variations in anti-tumor immune cell infiltration, antigen presentation capability, cytolytic activity, and immune checkpoint molecule expression. These results suggest that TAMs may serve as potential biomarkers for predicting immune responses in ACC patients.

### 3.6 M2 macrophages as biomarkers for poor lung metastasis prognosis in ACC

**Figure 6:**
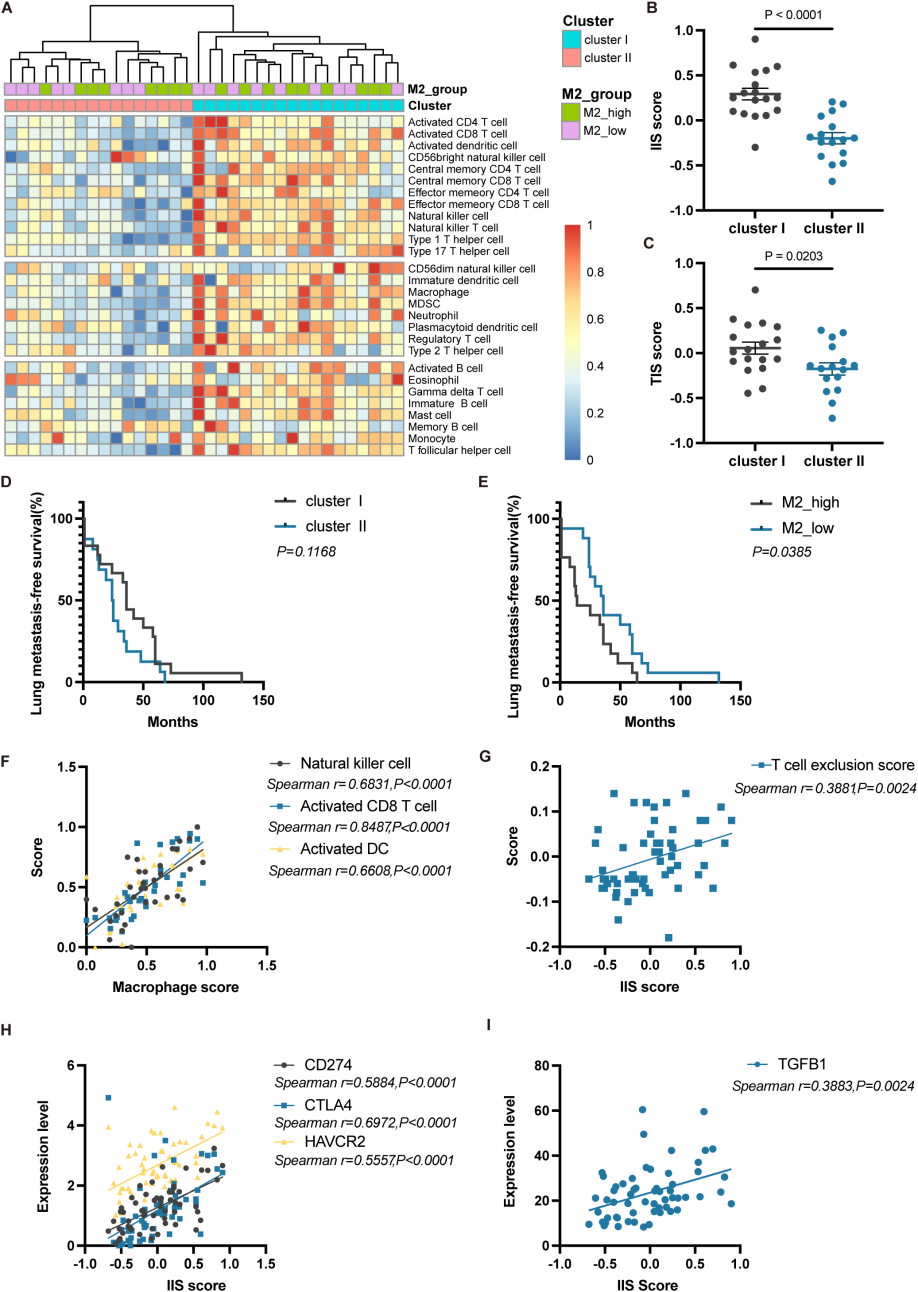
Immune phenotype and prognostic differences of lung metastases in ACC. **(A)** Heatmap showing unsupervised clustering analysis of lung metastasis samples based on 28 immune cell types. **(B)** Differences in IIS scores between Cluster I and Cluster II. **(C)** Differences in TIS scores between Cluster I and Cluster II. **(D)** Kaplan-Meier curves for lung metastasis-free survival between Cluster I and Cluster II. **(E)** Kaplan-Meier curves for lung metastasis-free survival between M2_high and M2_low groups. **(F)** Correlation analysis between macrophage scores and the scores of NK cell, activated CD8 T cell, and activated DC. **(G)** Correlation analysis between IIS scores and T cell exclusion scores. **(H)** Correlation analysis between IIS scores and the expression of CD274, CTLA4, and HAVCR2. **(I)** Correlation analysis between IIS scores and TGFB1 expression. The p values were calculated using the two-tailed Mann–Whitney U test in **(B, C)**, Log-rank test in **(D, E)** and Spearman correlation analysis in **(F–I)**. Data are presented as the mean ± SEM.

Due to the unique microenvironment of the lungs, circulating tumor cells (CTCs) of ACC interact with their surroundings, forming a metastatic niche distinct from the primary site. We next conducted a detailed analysis of ACC lung metastases. Unsupervised clustering of lung metastasis samples based on immune cell infiltration scores divided them into two clusters: Cluster I (immune cell-enriched) and Cluster II (immune cell-poor) (**Figure 6A**). Cluster I had significantly higher IIS and TIS compared to Cluster II (P<0.0001 and P<0.05, respectively) (**Figures 6B, C**). Differentially expressed genes between the two clusters were enriched in pathways related to immune response, chemokine signaling, T-cell activation, antigen processing and presentation, and primary immunodeficiency (**Supplementary Figures S4A-C**), implying that Cluster II may have defects in initiating immune responses and recruiting immune cells.

When comparing the lung metastasis-free survival between Cluster I and Cluster II, there was no significant difference (P=0.1168, Log-rank test) (**Figure 6D**). This indicates that even though Cluster I patients seemed to have a more favorable immune microenvironment, it did not translate into better metastasis-free survival. Due to the short follow-up period, no patients had reached the endpoint event, so overall survival differences between the two groups require longer follow-up to observe. Based solely on immune cell abundance, we did not observe prognostic differences in lung metastasis outcomes among ACC patients. Nevertheless, when stratifying patients based on the proportion of M2 macrophages into M2_high and M2_low groups, the M2_low group had significantly longer metastasis-free survival (P<0.05, Log-rank test) (**Figure 6E**). This suggests that high infiltration of M2 macrophages in tumors may be an indicator of early lung metastasis.

In lung metastases, activated CD8+ T cells, NK cells, and activated DCs were all positively correlated with macrophage scores (all P<0.0001) (**Figure 6F**), indicating that the infiltration of anti-tumor immune cells is often accompanied by a higher proportion of macrophages, thereby limiting their immune effects. Additionally, IIS scores were significantly positively correlated with T-cell exclusion scores (P<0.01) (**Figure 6G**). The expression of immune checkpoint molecules VSIR (P<0.0001), HAVCR2 (P<0.0001), LAG3 (P<0.0001), and the immunosuppressive cytokine TGFB1 (P<0.01) were also significantly positively correlated with IIS scores (**Figures 6H, I**). These results indicate that in lung metastasis patients with immune-enriched tumors, multiple immunosuppressive mechanisms may be present, including the enrichment of TAMs, T-cell exclusion, high expression of immune checkpoint molecules, and the production of immunosuppressive cytokines.

## 4 Discussion

In this study, we describe the tumor immune microenvironment landscape of primary adenoid cystic carcinoma and its pulmonary metastases. In malignant tumors, interactions between tumor cells, surrounding stromal cells, immune cells, and the extracellular matrix create a unique tumor heterogeneity. Distinct niches exist at different time points, spatial locations, and even within different regions of a single tumor, each characterized by unique microenvironments (25). The composition, function, spatial localization, and gene expression profiles of innate and adaptive immune infiltrates in the TIME often have established prognostic implications (47) and are associated with treatment resistance (48, 49). The primary cause of death in ACC patients is distant metastasis, particularly to the lungs. However, current treatments such as chemotherapy, targeted therapy, and ICIs like PD1 inhibitors, have shown disappointing results in metastatic ACC, underscoring the urgent need to develop effective therapies targeting distant metastases. Therefore, understanding the characteristics of the TIME in ACC metastatic sites is crucial for developing new therapeutic strategies.

Most current research on the TIME is limited to primary ACCs, with few studies analyzing the metastatic immune microenvironment. Cafferty et al.’s study included a small number of ACC metastatic samples and focused on the TIME differences among three invasive salivary gland carcinomas, without in-depth analysis of differences between primary ACCs and metastatic lesions (50). In this work, we collected primary ACC tumor samples and lung metastases. Through multi-omics analysis, we discovered the heterogeneity between the immune microenvironments of primary ACCs and lung metastases, filling the gap in research on the lung metastasis microenvironment of ACC. We found that the abundance of immune cell infiltration in both primary tumors and lung metastases was significantly lower than in adjacent normal tissues, suggesting that ACC generally remains an immune “cold” tumor, consistent with previous reports (51). However, compared to PT, L-MET had a higher degree of immune cell infiltration and stronger antigen-presenting capabilities. Contrary to expectations, the antitumor cytotoxic activity in L-MET was not higher than in PT. This indicates that lung metastases may possess additional immunosuppressive mechanisms that limit antitumor immune responses, facilitating the escape of CTCs from immune surveillance and allowing their colonization and growth in the lungs. Analyzing two common immune evasion mechanisms in tumors (35), we found that scores for both T cell dysfunction and T cell exclusion mechanisms were significantly higher in lung metastases. Additionally, L-MET had elevated degree of macrophage and immature dendritic cell infiltration. These findings correspond to a previous study that reported increased levels of macrophages, monocytic dendritic cells, and dysfunctional T cells in the pre-metastatic lung in the presence of a primary tumor, which forms a myeloid cell-rich immunosuppressive microenvironment (52). These results highlight the significant heterogeneity between primary and pulmonary metastatic lesions in ACC. Future preclinical studies of immunotherapies targeting ACC lung metastases should be conducted within appropriate organ-specific tumor microenvironments.

Macrophages represent a heterogeneous cell population known for their remarkable plasticity (53). They differentiate into various subtypes in response to different microenvironmental stimuli, such as tumor stroma and infected tissues. Functionally, macrophages are categorized into two subpopulations: classically activated macrophages (M1) and alternatively activated macrophages (M2) (53). M2 macrophages have been extensively reported to promote tumor progression and immune suppression in cancer. Our multi-omics analysis confirmed a notable enrichment of M2 macrophages in lung metastases of ACC. Another study on the immune microenvironment of breast cancer pulmonary metastases also revealed a considerable amplification of macrophages in lung metastatic sites (25). Additionally, the accumulation of suppressive macrophages at the invasive margins of lung metastases in melanoma and soft tissue sarcoma forms an immunosuppressive niche (25). These findings suggest that macrophage accumulation is not unique to ACC lung metastases but may be a common feature of the pulmonary metastatic microenvironment.

Through single-cell sequencing, we identified multiple macrophage subpopulations in ACC. Tumor-infiltrating macrophages were predominantly of the M2 type, with high expression of SPP1. SPP1 encodes osteopontin, a phosphoprotein that mediates interactions between TAMs and tumor cells, with high expression linked to poorer survival outcomes (42). In the adjacent primary lesions and lung tissues, various macrophage populations were identified. Cluster 1 showed high expression of chemokines CCL22 and CCL17, which recruit immunosuppressive cells such as Th2 cells (54). MARCO, a pattern recognition receptor highly expressed in Cluster 2, is involved in pathogen recognition and clearance, but MARCO-expressing TAMs can inhibit the activation and proliferation of cytotoxic T cells and NK cells, as well as cytokine production (44). Clusters 3 and 4 showed high expression of the inhibitory receptor LILRB5, which suppresses macrophage activation and function, reducing inflammation and cytokine secretion (55). This regulatory function helps maintain immune system balance, preventing excessive immune responses and protecting tissues from inflammatory damage. However, in pathological conditions such as cancer, this may play a critical role in immune evasion, helping tumor cells avoid detection and attack by the immune system. These findings indicate that many of these genes are related to the immunoregulatory functions of M2 macrophages. The presence of a substantial number of immunosuppressive macrophages in the tumor and its surrounding microenvironment may significantly contribute to immune evasion and resistance to immunotherapy in ACC.

TAMs are a major component of the TIME and significantly affect the efficacy of immune checkpoint inhibitors (56). Eliminating the pre-existing immunosuppressive environment in the TIME can help overcome primary resistance in cancer patients and enhance the therapeutic efficacy of ICIs (38). Our comprehensive analysis of the immune microenvironment in ACC pulmonary metastases revealed the central role of TAMs in this context. By grouping ACC patients based on macrophage transcription levels, we observed differences in their immune landscapes and lung metastases free survival. High infiltration of M2 macrophages was closely associated with early pulmonary metastasis in patients. A study on colorectal cancer confirmed that TAMs promote tumor metastasis through derived extracellular vesicles (57). These findings suggest that TAMs could serve as potential biomarkers for predicting the immune therapy response and prognosis of pulmonary metastasis in ACC. However, it is necessary to validate the predictive effectiveness of TAMs for the immune therapy response in pulmonary metastatic ACC in real clinical cohorts. Targeting TAMs represents a promising anticancer strategy. By eliminating or reprogramming TAMs from an M2 pro-tumor state to an M1 anti-tumor state, unexpected benefits may be achieved for patients with pulmonary metastatic ACC.

However, our current study is based on the transcriptomic analysis of the TIME in available samples, which has certain limitations. First, due to the difficulty of obtaining lung metastasis samples, our study had a relatively small sample size, potentially limiting the generalizability of the findings. Second, the transcriptional characteristics should be validated in larger patient cohorts through immunohistochemistry, assessing various markers that characterize NK cells, B cells, dendritic cells, and macrophage subpopulations, among others. It’s not just the number of immune cells that matters—their spatial distribution often plays a critical role as well. Therefore, future applications of spatial transcriptomics and proteomics may yield further insights. Moreover, anti-cancer treatments can alter the tumor immune microenvironment. Given that there is no standard treatment for distant metastases of ACC, many patients in the lung metastasis cohort had already received multiple therapies, including but not limited to chemotherapy, targeted therapy, and immunotherapy, by the time the samples were collected. These systemic treatments may have affected the TIME in the metastases. Thus, the unique characteristics of the lung metastasis microenvironment may result from a combination of organ-specific factors and the effects of systemic treatment. However, our study did not explore the influence of different treatment modalities on the tumor immune microenvironment in depth. The mechanisms underlying the formation of the lung metastasis microenvironment in ACC, and the role TAMs play within it, require further investigation using a variety of *in vitro* and *in vivo* models.

## 5 Conclusion

In this research, we characterized the TIME of head and neck primary and lung metastatic ACC, uncovering unique changes in immune composition at the metastatic niche and highlighting the heterogeneity between primary and metastatic lesions. Our results provide a deeper understanding of the immune microenvironment in lung metastases of ACC and underscore the critical role of macrophages in creating an immunosuppressive environment. These insights may inform new therapeutic strategies targeting specific macrophage populations within the metastatic niche. Understanding organ-specific immune changes is essential for developing precise and effective immunotherapies to suppress metastatic recurrence. Future immunotherapy for ACC patients should consider the distinct immune microenvironment of metastatic sites.

## Data Availability

The original contributions presented in the study are publicly available. This data can be found here: Gene Expression Omnibus (GEO). The GEO number is GSE282732.
