# Supplementary material for: Heterogeneous immune landscapes and macrophage dynamics in primary and lung metastatic adenoid cystic carcinoma of the head and neck
Source: Front Immunol. 2024 Dec 4;15:1483887. doi: 10.3389/fimmu.2024.1483887 (PMC11653016; doi:10.3389/fimmu.2024.1483887)
Supplement: Supplementary file 1 [file Presentation1.zip › Data Sheet 1/ Supplementary material presentation/Supplementary Figure Legends.docx]

**Supplementary Figure Legends**

**Supplementary Figure 1. (A)** Heatmap of 28 immune cell scores comparing lung metastases and primary ACCs. **(B)** Correlation analysis among tumor-infiltrating immune cells.

**Supplementary Figure 2. (A)** Heatmap of HLA antigen presentation-related gene expression. **(B)** Statistical plots of mRNA expression levels for HLA-A, HLA-B, HLA-C, HLA-DRA, HLA-DPA1, and HLA-DQA1 between primary ACCs and lung metastases.

**Supplementary Figure 3. (A)** Stacked bar charts of immune cell deconvolution in 3 pairs of primary tumors and matched lung metastases. **(B)** Correlation of M1 macrophages with CD8 T cells and activated DCs.

**Supplementary Figure 4. (A)** Volcano plot of differentially expressed genes between Cluster I and Cluster II. KEGG **(B)** and GO-BP **(C)** enrichment analysis bubble plots based on differentially expressed genes between Cluster I and Cluster II.
