# Supplementary figures and images for: Heterogeneous immune landscapes and macrophage dynamics in primary and lung metastatic adenoid cystic carcinoma of the head and neck
Source: Front Immunol. 2024 Dec 4;15:1483887. doi: 10.3389/fimmu.2024.1483887 (PMC11653016; doi:10.3389/fimmu.2024.1483887)

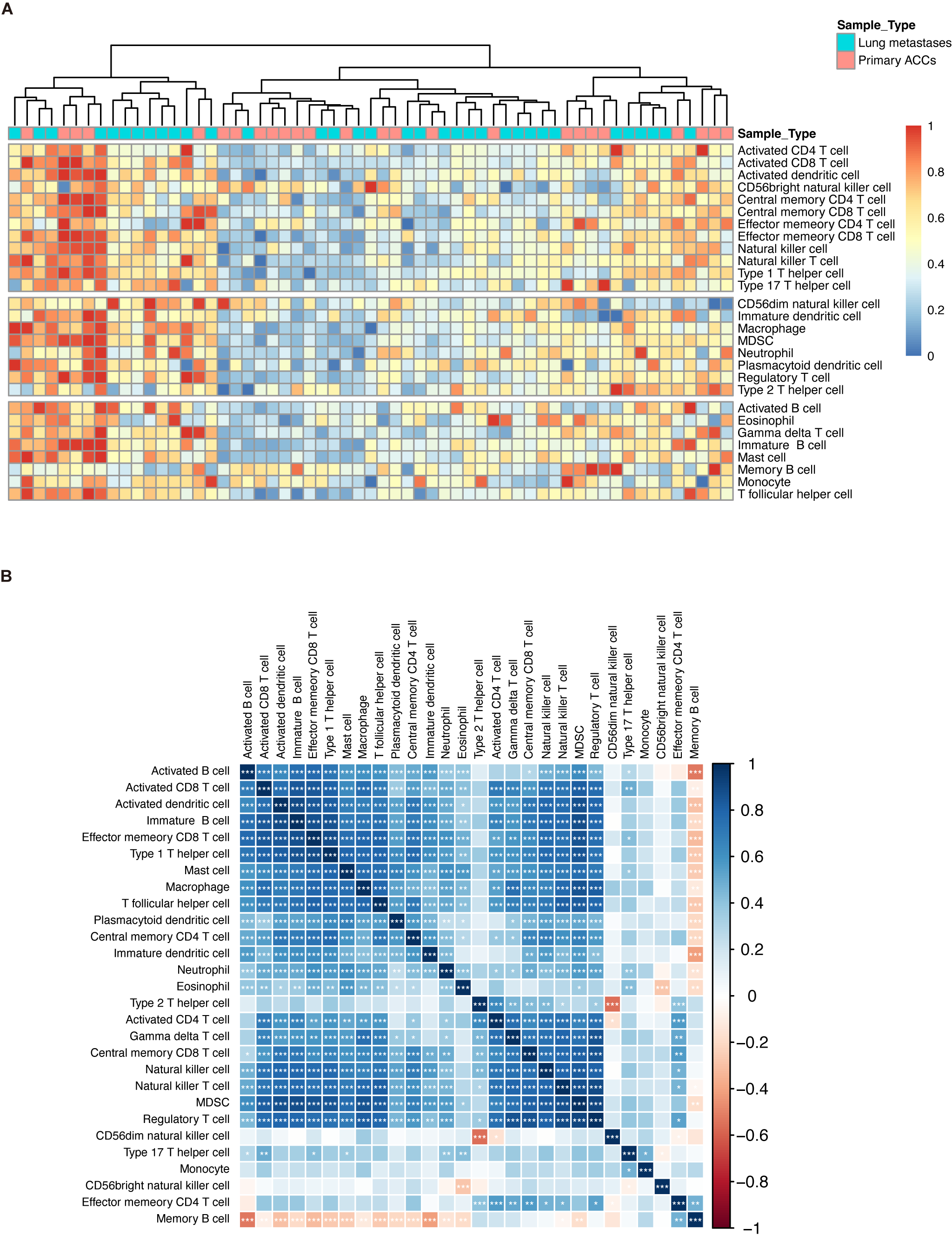

Supplement: Supplementary file 1 [file Presentation1.zip › Data Sheet 1/ Supplementary material presentation/Supplementary Figure 1.tif]

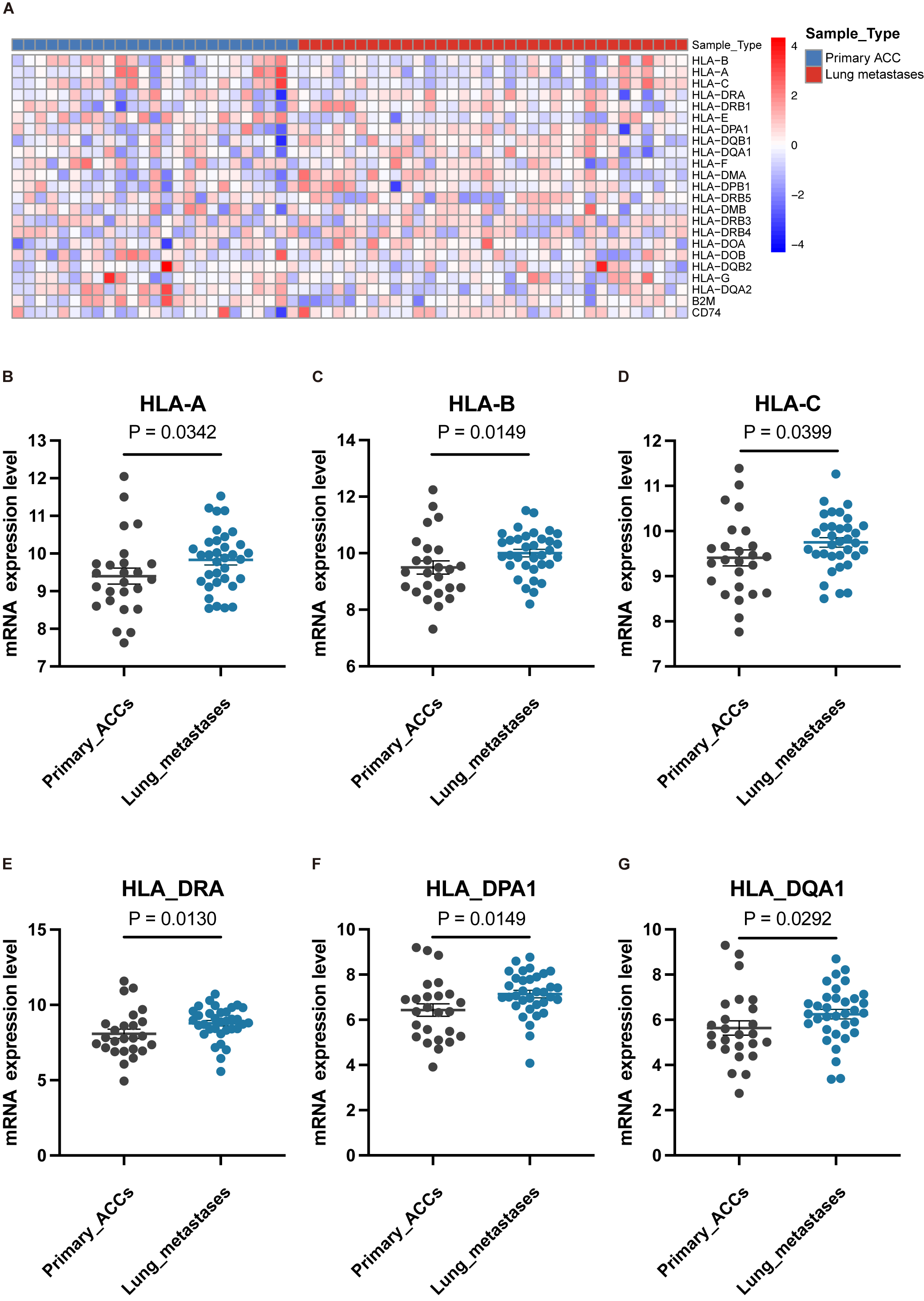

Supplement: Supplementary file 1 [file Presentation1.zip › Data Sheet 1/ Supplementary material presentation/Supplementary Figure 2.tif]

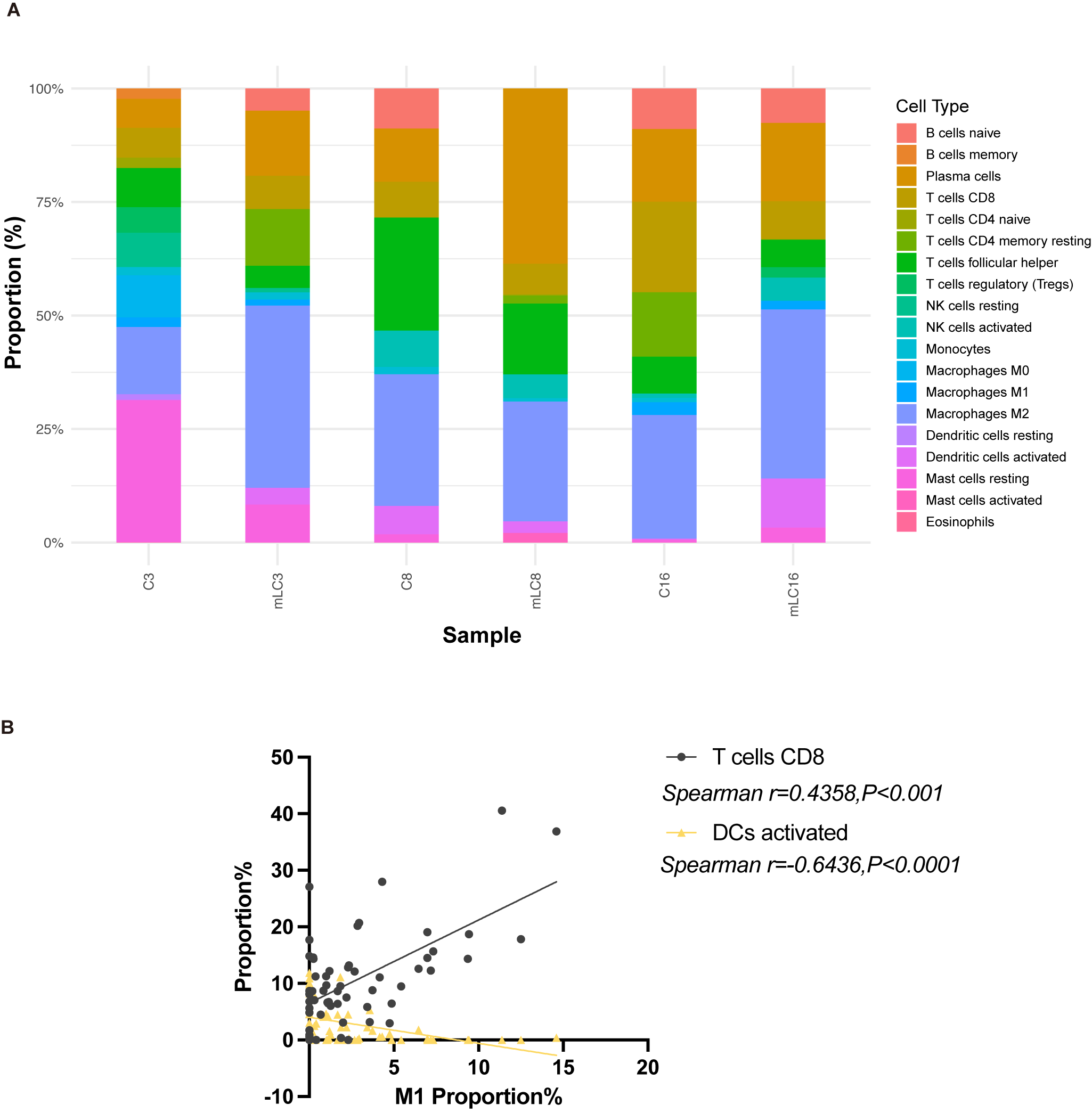

Supplement: Supplementary file 1 [file Presentation1.zip › Data Sheet 1/ Supplementary material presentation/Supplementary Figure 3.tif]

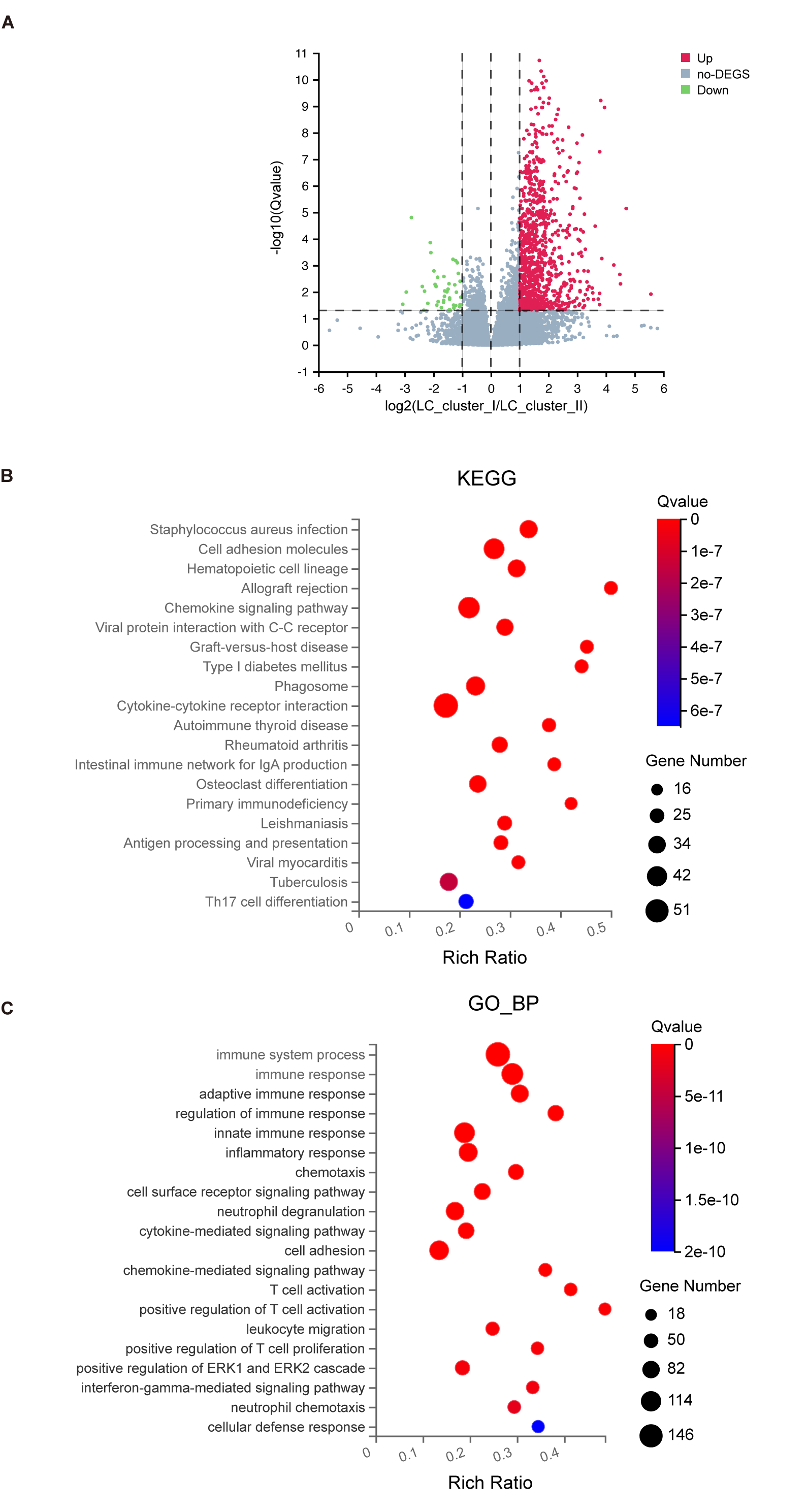

Supplement: Supplementary file 1 [file Presentation1.zip › Data Sheet 1/ Supplementary material presentation/Supplementary Figure 4.tif]
